# Clinical relevance of integrin alpha 4 in gastrointestinal stromal tumours

**DOI:** 10.1111/jcmm.13502

**Published:** 2018-01-29

**Authors:** Olli‐Pekka Pulkka, John‐Patrick Mpindi, Olli Tynninen, Bengt Nilsson, Olli Kallioniemi, Harri Sihto, Heikki Joensuu

**Affiliations:** ^1^ Laboratory of Molecular Oncology Translational Cancer Biology Program Department of Oncology University of Helsinki Helsinki Finland; ^2^ Institute for Molecular Medicine Finland (FIMM) University of Helsinki Helsinki Finland; ^3^ Department of Pathology University of Helsinki and HUSLAB Helsinki University Hospital Helsinki Finland; ^4^ Sahlgrenska University Hospital Gothenburg Sweden; ^5^ Science for Life Laboratory Department of Oncology & Pathology Karolinska Institutet Stockholm Sweden; ^6^ Comprehensive Cancer Center Helsinki University Hospital Helsinki Finland

**Keywords:** gastrointestinal stromal tumour, ITGA4, survival, metastasis, proliferation, invasion

## Abstract

The molecular mechanisms for the dissemination and metastasis of gastrointestinal stromal tumours (GIST) are incompletely understood. The purpose of the study was to investigate the clinical relevance of integrin alpha 4 (ITGA4) expression in GIST. GIST transcriptomes were first compared with transcriptomes of other types of cancer and histologically normal gastrointestinal tract tissue in the MediSapiens *in silico* database. *ITGA4* was identified as an unusually highly expressed gene in GIST. Therefore, the effects of *ITGA4* knock‐down and selective integrin alpha 4 beta 1 (VLA‐4) inhibitors on tumour cell proliferation and invasion were investigated in three GIST cell lines. In addition, the prognostic role of ITGA4 expression in cancer cells was investigated in a series of 147 GIST patients with immunohistochemistry. Inhibition of ITGA4‐related signalling decreased GIST cell invasion in all investigated GIST cell lines. ITGA4 protein was expressed in 62 (42.2%) of the 147 GISTs examined, and expression was significantly associated with distant metastases during the course of the disease and several adverse prognostic features. Patients whose GIST expressed strongly ITGA4 had unfavourable GIST‐specific survival and overall survival compared to patients with low or no ITGA4 expression. Taken together, ITGA4 is an important integrin in the molecular pathogenesis of GIST and may influence their clinical behaviour.

## Introduction

GISTs are one of the most common types of soft‐tissue sarcoma. Gain of function mutations in *KIT* (encodes the KIT receptor tyrosine kinase) is major molecular drivers in GISTs 1. Approximately 75% of GISTs harbour mutated *KIT* 2 and one‐third of GISTs that lack *KIT* mutation contain an activating mutation in the platelet‐derived growth factor receptor α (*PDGFRA*) gene 3. About 20% of GISTs are metastatic at the time of the diagnosis 4. Metastases occur most often in the liver and other intra‐abdominal sites, whereas metastases outside of the abdomen are uncommon 5, 6, 7. Imatinib mesylate is the standard first‐line therapy for patients with inoperable or metastatic GIST 8 and the standard adjuvant therapy after surgery for patients with a high estimated risk for recurrence 9.

Although *KIT* and *PDGFRA* mutations are likely of key importance in the molecular pathogenesis of GISTs, identical single *KIT* or *PDGFRA* mutations may be associated with widely different tumour mitotic counts and GIST patient survival outcome 2. Such findings suggest that aberrations in several other genes may be crucially important in determining the propensity of GIST cells to proliferate, invade and to give rise to metastases.

Relatively little is known about the relevance of integrins in the molecular pathogenesis and clinical behaviour of GISTs. Integrins have multiple roles in the cell signalling and in the regulation of cell growth, division, survival, differentiation, migration and apoptosis 10. Integrin activity may change the polarity of migrating cells and the assembly of the extracellular matrix that may influence cancer metastasis 11. Integrin‐targeting drugs are under clinical investigation for several diseases including cancer 12, 13, and many of this target the RGD‐binding integrins 12. Besides cell migration and invasion, integrins may also control cell proliferation 13. Adhesion‐dependent control of cell proliferation may be deregulated in cancer, and integrins regulate the growth of some cancers 14, 15. Increased expression of αvβ3, αvβ5, α5β1, α6β4, α4β1 and αvβ6 integrins has been linked with cancer progression 12.

The ITGA4 family of integrins mediate cell–cell adhesions that are crucial especially to the immune function 16. The α4 peptide (CD49d) associates with either the β1 chain (CD29) or the β7 chain forming α4β1 (very late antigen‐4, VLA‐4) and α4β7 (lymphocyte Peyer patch adhesion molecule) integrins, respectively. Alpha 4 integrins are involved in haematopoiesis, myogenesis, and cardiac and placental development 17, 18, 19. The alpha 4 integrins are involved also in the surveillance, inflammation and pathogenesis of cardiovascular diseases 20. α4β1 binds to the vascular cell adhesion molecule‐1 (VCAM‐1) that is expressed on the surface of endothelial and stromal cells and to fibronectin in the extracellular matrix, whereas α4β7 binds to the mucosal vascular addressin cell adhesion molecule‐1 (MAd‐CAM‐1) 11. Natalizumab, a humanized monoclonal antibody that targets ITGA4 has been approved by the U.S. Food and Drug Administration (FDA) for the treatment of multiple sclerosis and Crohn's disease 21, 22.

The role of ITGA4 is unknown in GISTs. In this study, we report that *ITGA4* is often expressed strongly in GISTs compared to many other cancers and histopathologically normal human tissues, suggesting a molecular pathologic role for ITGA4 in GIST. We found further that high GIST ITGA4 expression in the tumour cells is associated with unfavourable prognosis of patients and undertook functional studies to investigate whether inhibition of ITGA4 with siRNA or two VLA‐4‐specific inhibitors prevents invasion of GIST cells. As ITGA4 can be targeted with monoclonal antibodies such as natalizumab, and VLA‐4‐specific inhibitors such as BIO1211 and BIO5192, these findings suggest that ITGA4 may be a potential therapeutic target in GIST.

## Materials and methods

### Patients

ITGA4 protein expression was determined in 173 soft‐tissue sarcomas consisting of eight different histopathological types. Formalin‐fixed paraffin‐embedded (FFPE) tissue samples were selected at random and collected from the archives of the Department of Pathology, Helsinki University Hospital. We selected at random 13 GISTs, 29 leiomyosarcomas, eight synovial sarcomas and nine undifferentiated pleomorphic sarcomas from these 173 sarcomas for the quantitative PCR (qPCR) analysis of tumour *ITGA4* mRNA content.

The associations between tumour ITGA4 expression, patient features, and GIST clinical and histopathological parameters were investigated from another series that consisted of 288 patients diagnosed with KIT‐positive GIST in western Sweden from 1983 through 2000 6, 23. The characteristics of the patients and the GISTs in this series are described in detail elsewhere 6. These patients had undergone surgery for GIST, and none received imatinib or other tyrosine kinase inhibitors as adjuvant systemic treatment for GIST after surgery. GIST tissue was available for the original tissue microarray (TMA) block from 241 (83.7%) of the 288 patients 24, of whom we included in this study, all 147 (61.0%) patients whose gender, tumour diameter, tumour site in the gastrointestinal tract, and follow‐up data were available, and whose GIST diagnosis had been verified at a histopathological review. The stratification for the risk of GIST recurrence was performed according to the U.S. National Institutes of Health (NIH) criteria 25. Institutional review boards of the Helsinki University Hospital and the Gothenburg University approved the study.

### Outlier gene expression analysis

Genes that are highly expressed in GIST in comparison with histologically normal gastrointestinal tract tissue samples were identified and ranked using the gene tissue index (GTI) outlier statistics as described elsewhere 26. Cancer and normal tissue mRNA expression data were extracted from the MediSapiens *in silico* database that consists of transcriptomes of 17,330 human genes across 9783 human tissue samples and 175 histologically different tissue types (available at ist.medisapiens.com) 27. The analysis of *ITGA4* mRNA expression in the database was based on six GISTs and 34 histologically normal gastrointestinal tract tissue samples.

### Immunohistochemistry

For the construction of the TMA, representative areas consisting of tumour were identified from each FFPE tissue block on H&E‐stained tissue sections, and two 0.7 mm (the sarcoma series) or one 0.6 mm diameter (the western Sweden series) tumour tissue biopsies were subsequently taken. The histological features of the tissue selected for the western Sweden TMA were assessed by a pathologist, and the validity of the tissue was confirmed by requiring positive staining for KIT at immunohistochemical evaluation of the TMA.

**Figure 1:**
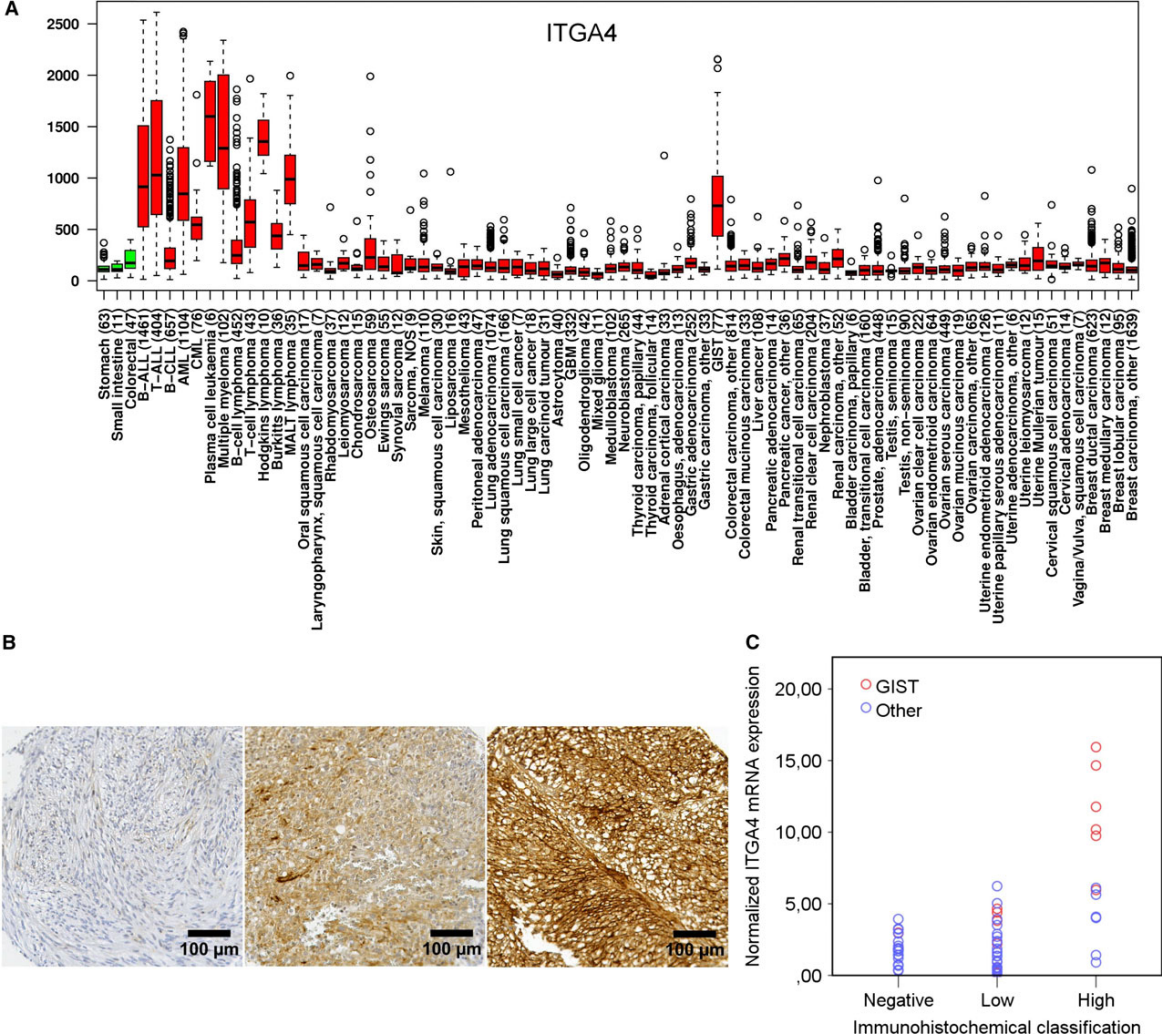
(**A**) A box–whisker plot showing the relative *ITGA4* mRNA expression in histopathologically normal gastric, small intestine and colorectal tissue (green boxes) and in different types of cancer (red boxes). The number of samples studied is indicated in the brackets. (**B**) Examples of negative, low and high ITGA4 expression in GIST tissue samples (magnification ×200). (**C**) Association between ITGA4 protein expression at immunohistochemistry and the normalized *ITGA4* mRNA expression (*P* < 0.001) determined from the same samples with qPCR. Panel A is modified from IST Online™ (ist.medisapiens.com).

ITGA4 protein expression was evaluated from 5‐μm TMA sections using immunohistochemistry. The TMA slides were deparaffinized in xylene and hydrated in a graded series of alcohol. Endogenous peroxide activity was blocked with hydrogen peroxide incubation. Antigen retrieval for ITGA4 was carried out in sodium citrate (10 mmol/l, pH 6.0) in an autoclave (at 120°C for 2 min.). The primary antibody was a polyclonal rabbit ITGA4 antibody (dilution 1:100; A9385; LifeSpan BioSciences, Seattle, WA, USA), which was diluted in a PowerVision pre‐antibody blocking solution and incubated for 30 min. on the slides at room temperature. Primary antibody binding was detected using a BrightVision+ Histostaining kit (Immunologic BV, Duiven, The Netherlands) and 3,3′‐diaminobenzidine (ImmPACT^™^ DAB; Vector Laboratories, Burlingame, CA, USA) following the manufacturer's instructions. The slides were counterstained with Mayer's haematoxylin. ITGA4 showed uniform staining within 13 large GIST tissue sections, and, therefore, tumour ITGA4 expression was categorized as negative, low or high based on the staining intensity (Fig. 1B). Only cancer cell ITGA4 expression was scored, but the endothelial cells and leucocytes were also observed to stain positive for ITGA4. The immunostainings were assessed blinded without knowledge of the clinical or histopathological data.

### qPCR assay

RNA was extracted from the FFPE tissue sections using a High Pure RNA Paraffin kit (Roche Diagnostics GmbH, Mannheim, Germany) and from the GIST cell lines using a High Pure RNA Isolation Kit (Roche Diagnostics GmbH). The mRNA was reverse transcribed to cDNA with a SuperScript^®^ VILO^™^ cDNA Synthesis Kit (Invitrogen, Carlsbad, CA, USA) according to the manufacturer's instructions.

*ITGA4* mRNA expression of 13 GISTs, 29 leiomyosarcomas, eight synovial sarcomas and nine undifferentiated pleomorphic sarcomas was quantitated with real‐time qPCR using hydrolysis probes (*i.e*. hybridization probes labelled with a reporter dye and a quenching dye) in a LightCycler 480 instrument (Roche Diagnostics GmbH). cDNA was amplified in a 20 μl PCR mixture using LightCycler 480 Probes Master reagents (Roche Diagnostics GmbH) and a fluorescein‐labelled locked nucleic acid (LNA) hydrolysis probe 13 or an LNA hydrolysis probe G6PD from a Universal ProbeLibrary Set (Roche Diagnostics GmbH). The PCR mixture contained 1× PCR buffer, 100 nmol/l of probe and 200 nmol/l of each primer specific for the ITGA4‐coding region (forward: 5′‐AGGAAGTTCCAGGTTACATTGTTT‐3′; reverse: 5′‐TTAGAAGAGAAATAGAATCTTGGTGGA‐3′) or for *G6PD* as the reference DNA (forward: 5′‐GAGCCAGATGCACTTCGTG‐3′; reverse: 5′‐GGGCTTCTCCAGCTCAATC‐3′). The ProbeFinder program at the Assay Design Center of Universal ProbeLibrary (www.universalprobelibrary.com; Roche Diagnostics GmbH) was used for the design of the primers and the probes. The cycling parameters consisted of an initial denaturation at 95°C for 10 min., followed by 60 cycles with denaturation at 95°C for 15 sec., annealing at 60°C for 45 sec. and elongation at 72°C for 45 sec. The results were analysed using the Basic Relative Quantification method (Roche Diagnostics GmbH).

### Cell lines

GIST882 and GIST48 cell lines were a kind gift from Dr. Jonathan A. Fletcher (Harvard Medical School, Boston, MA, USA). GIST‐T1 cell line was purchased from Cosmo Bio (Tokyo, Japan). GIST882 is a primary human GIST cell line with a homozygous missense mutation in *KIT* exon 13, encoding a p.K642E mutant *KIT* oncoprotein 28. GIST48 is a GIST cell line that progressed after an initial response to imatinib and harbours a primary *KIT* exon 11 p.V560D missense mutation and a secondary exon 17 missense mutation p.D820A 29. The GIST‐T1 cell line was established from a metastatic pleural tumour from a primary gastric GIST. GIST‐T1 is a *KIT* exon 11 mutant cell line that has a heterozygous in‐frame deletion of 57 bases 30. The authenticity of the GIST882, GIST48 and GIST‐T1 cell lines was confirmed with DNA sequencing. The cells were cultured in a humidified 5% CO_2_ atmosphere at 37°C. GIST882 and GIST48 cells were cultured in the RPMI 1640 medium (GIBCO, Carlsbad, CA, USA), supplemented with 20% foetal bovine serum with 2% penicillin/streptomycin (GIBCO), and GIST‐T1 cells were cultured in a DMEM medium (Lonza, Walkersville, MD, USA) supplemented with 10% foetal bovine serum with 2% penicillin/streptomycin (GIBCO).

### VLA‐4 inhibitors

BIO1211 and BIO5192, both integrin α4β1 (VLA‐4)‐specific inhibitors, were purchased from Tocris Bioscience (Bristol, UK). BIO1211 was reconstituted in PBS and BIO5192 was in DMSO.

### Cell proliferation and invasion assays

GIST cell proliferation was studied with the 3‐(4,5‐dimethylthiazol‐2‐yl)‐2,5‐diphenyltetrazolium bromide (MTT) assay (Roche Diagnostics, Indianapolis, IN, USA). For the assay, 15,000 GIST882 cells, 20,000 GIST48 cells and 5000 GIST‐T1 cells were plated in 96‐well plates. The analyses were performed three times, and eight replicate wells were assessed at each time‐point. The effect of siRNAs for cell proliferation was analysed at 0, 24, 48, 72 and 98 hrs, and the effect of VLA‐4 inhibitors was analysed at 72 hrs using 10 different concentrations. After adding 10 μl of the MTT reagent into each well and incubation for 4 hrs at 37°C, a solubilization solution was added to dissolve the formazan crystals overnight at 37°C. The 96‐well plates were read with a Multiscan EX Microplate photometer (Thermo Scientific, Rockford, IL, USA) at the wavelength of 540 nm.

For the cell invasion assay, 50 μl of 2.5 mg/ml matrigel containing 5 μg/ml fibronectin was placed into the upper chamber of a 24‐well transwell (Corning^™^ Falcon^™^ Cell Culture Inserts, 8.0 μm pore size; Fisher Scientific). Six hundred microlitre of the RPMI 1640 medium supplemented with 20% foetal bovine serum and 2% penicillin/streptomycin was added to the lower chamber of the transwell. GIST882 cells were plated at the density of 20,000 cells/well, GIST48 cells at the density of 30,000 cells/well and GIST‐T1 cells at the density of 10,000 cells/well in a medium without serum to the upper chamber of the transwell. GIST48 and GIST882 cells were incubated for 24 hrs and GIST‐T1 cells for 96 hrs. Cell invasion was measured in three separate experiments by counting the cells within 10 photographed fields of the microscope (Leica CTR6000; Leica microsystems, Bannockburn, IL, USA; magnification ×200). The number of invaded cells was expressed as the average number of invaded cells per one microscope field.

### siRNA transfections

The Lipofectamine 2000 Transfection Reagent (Invitrogen) was used for the GIST cell siRNA transfections. The cells were first serum starved for 6 hrs in an Opti‐MEM reduced serum medium (GIBCO). Following this, transfections were performed according to the manufacturer's instructions adding 5 pmol/l of siRNA onto the GIST882 and GIST48 cells and 10 pmol/l of siRNA onto the GIST‐T1 cells. The transfections were performed with an ON‐TARGET plus Human KIT siRNA and an ON‐TARGET plus Human ITGA4 siRNA, which are pools of target‐specific siRNAs (Thermo Scientific Dharmacon, Rockford, IL, USA). The ON‐TARGET plus Non‐Targeting Pool (Thermo Scientific Dharmacon) was used as the negative control.

### Western blotting

Cultured GIST cell lines were rinsed in PBS (Lonza) and scraped on ice into a RIPA lysis buffer (Pierce Biotechnology, Rockford, IL, USA) containing a Pierce Protease and Phosphatase Inhibitor Tablet (Thermo Fisher Scientific Inc.), followed by sonication. The protein concentrations were measured using a Pierce BCA Protein Assay Kit (Thermo Fisher Scientific Inc.). Ten microgram of protein was separated using gel electrophoresis and blotted onto an Immobilon‐P Transfer Membrane (Millipore, Billerica, MA, USA). The primary antibodies were a monoclonal rabbit anti‐ITGA4 antibody (dilution 1:1000; clone D2E1; Cell Signaling Technology, Danvers, MA, USA), a polyclonal rabbit anti‐c‐KIT antibody (dilution 1:10,000; A4502; Agilent Technologies Dako, Glostrup, Denmark), a polyclonal rabbit anti‐Phospho‐c‐KIT (Tyr703) antibody (dilution 1:10,000; 3391; Cell Signaling Technology), a polyclonal rabbit β‐Actin (dilution 1:10,000; Bethyl Laboratories, Montgomery, TX, USA) and a peroxidase‐conjugated AffiniPure Goat Anti*‐Rabbit* antibody was used as the secondary antibody (dilution 1:10,000; Jackson Immuno Research, West Grove*,* PA, USA). Blot immunostains were treated with a SuperSignal West Pico Chemiluminescent Substrate (Thermo Fisher Scientific Inc.), exposed to an X‐ray film, and the films were developed with a Kodak Medical X‐ray Processor 102 (Eastman Kodak; Rochester, NY, USA).

### Statistical analysis

The frequency tables were analysed with the chi‐squared test. Continuous parameters were compared with the Mann–Whitney test or the Kruskal–Wallis test. Cumulative survival was estimated with the Kaplan–Meier method and the log‐rank test. Overall survival was calculated from the date of the GIST diagnosis to the date of death, censoring patients who were alive on the last date of follow‐up. GIST‐specific survival was computed from the date of the diagnosis to death deemed to result from GIST, censoring patients who died from another cause on the date of death and patients who were alive on the last date of follow‐up. All *P*‐values are two‐sided. The inter‐rater agreement in immunohistochemical scoring of ITGA4 between two independent raters (O.P.P. and O.T.), and the intra‐rater agreement were measured by computing Cohen's kappa coefficient. The statistical calculations were carried out using an SPSS Statistics package v. 22.0 (IBM, Armonk, NY, USA).

## Results

### ITGA4 expression in cancer

*ITGA4* mRNA expression was high in GISTs as compared with other types of cancer and healthy gastrointestinal tract tissues available in the MediSapiens database (IST Online^™^ at ist.medisapiens.com) based on the GTI outlier analysis (Fig. 1A). Only GISTs and haematological malignancies expressed high levels of *ITGA4* mRNA. For comparison, in a similar analysis, *KIT* mRNA turned out to be highly expressed in GISTs as compared with the reference group (Fig. S1).

Expression of the ITGA4 protein was next investigated with immunohistochemistry on a sarcoma TMA. ITGA4 was detected in 44 (78.6%) of the 56 GISTs examined, of which expression was classified as high in 16 (28.6%) and low in 28 (50.0%; Fig. 1B and Table 1). ITGA4 expression (either low or high) was detected also in other types of sarcomas, but much less frequently than in GISTs (61 [52.1%] out of 117, *P* = 0.0009). Similarly, high tumour ITGA4 expression was also more frequent in GISTs in comparison with other sarcomas examined (16 [28.6%] out 56 *versus* 16 [13.7%] out of 117, *P* = 0.018). Besides GIST, strong ITGA4 expression was present frequently in four (10.5%) of 38 undifferentiated pleomorphic sarcomas, in eight (26.7%) of 30 leiomyosarcomas and four (23.5%) of 17 synovial sarcomas.

**Table 1:** ITGA4 expression in 173 human sarcomas

Tumour type	No. of tumours	ITGA4 expression		
		Negative *n* (%)	Low *n* (%)	High *n* (%)
Gastrointestinal stromal tumour (GIST)	56	12 (21.4)	28 (50.0)	16 (28.6)
Angiosarcoma	11	4 (36.4)	7 (63.6)	0 (0.0)
Chondrosarcoma	8	6 (75.0)	2 (25.0)	0 (0.0)
Fibrosarcoma	5	5 (100.0)	0 (0.0)	0 (0.0)
Undifferentiated pleomorphic sarcoma	38	21 (55.3)	13 (34.2)	4 (10.5)
Leiomyosarcoma	30	7 (23.3)	15 (50.0)	8 (26.7)
Liposarcoma	8	4 (50.0)	4 (50.0)	0 (0.0)
Synovial sarcoma	17	9 (52.9)	4 (23.5)	4 (23.5)
Total	173	68 (39.3)	73 (42.2)	32 (18.5)

The specificity of ITGA4 immunostaining was examined by comparing ITGA4 protein expression at immunohistochemistry with *ITGA4* mRNA expression measured with qPCR. Thirteen randomly selected GISTs, 29 leiomyosarcomas, eight synovial sarcomas and nine undifferentiated pleomorphic sarcomas were included in the analysis. Of these 59 tumours, six GISTs, five leiomyosarcomas and one synovial sarcoma showed high ITGA4 expression at immunohistochemistry. Weak ITGA4 expression was present in five GISTs, 16 leiomyosarcomas, two synovial sarcomas and three undifferentiated pleomorphic sarcomas. The median *ITGA4* gene to control *G6PD* gene mRNA expression ratio in GIST was 6.7 (range = 0.18–15.94, *P = *0.002) and in other tumours 2.1 (range = 0.10–6.23). ITGA4 protein expression at immunohistochemistry correlated significantly with *ITGA4* mRNA expression at qPCR lending support to the specificity of the immunohistochemical staining (*P* < 0.001, Fig. 1C).

### Clinical relevance

The clinical relevance of GIST ITGA4 protein expression was investigated in the western Sweden population‐based GIST patient series. As the current study is based on a subset of the patients in the western Sweden series, we compared age at the time of the diagnosis, gender, the NIH risk classification distribution, and the main GIST prognostic features in the current subset and the entire western Sweden series. We found no significant differences between the current subset and the entire series in any of these parameters, suggesting that no major imbalance had occurred in patient selection (Table S1).

The median age of the patients at the time of GIST diagnosis was 70 years (range, 30–92 years), and 74 (50.3%) of the patients were male. GIST ITGA4 expression was present in 62 (42.2%) of the 147 GISTs. High expression was present in 14 (9.5%) GISTs, low expression in 48 (32.7%), and 85 (57.8%) tumours were negative in immunostaining for ITGA4. When GISTs with negative, low or high ITGA4 expression were compared, ITGA4 expression was associated with a tumour location in the stomach, the high NIH risk stratification group for recurrence, presence of tumour necrosis, and a high tumour mitotic count (all *P*‐values <0.05; Table 2). GIST ITGA4 expression was associated also with presence of distant metastases during the course of the disease (*P* = 0.001). The majority of the 20 GISTs with metastases present at the time of GIST diagnosis were ITGA4‐positive (negative *n* = 7, weak *n* = 10, high *n* = 3), although this association between GIST ITGA4 expression and metastases was not significant (*P* = 0.084). GIST ITGA4 expression was not significantly associated with tumour size when size was tested as a continuous variable (Table 2). Similarly, when small GISTs (<2 cm in diameter) were compared with larger GISTs, there was no difference in GIST ITGA4 expression between the groups. When the patients with GIST with either negative or low ITGA4 expression were combined and compared to those with high ITGA4 expression, the results remained similar (Table S2).

**Table 2:** Associations between GIST ITGA4 expression and nine clinicopathological factors in the western Sweden patient series

Factor	GIST ITGA4 expression			*P*
	Negative *N* = 85 *n* (%)	Low *N* = 48 *n* (%)	High *N* = 14 *n* (%)	
Gender				
Female	46 (54.1)	21 (43.8)	6 (42.9)	0.448
Male	39 (45.9)	27 (56.2)	8 (57.1)	
Location				
Gastric	37 (43.5)	31 (64.6)	10 (71.4)	0.023
Non‐gastric	48 (56.5)	17 (35.4)	4 (28.6)	
NIH risk stratification				
Low/intermediate	62 (43.5)	28 (73.7)	4 (36.4)	0.009
High	16 (56.5)	10 (26.3)	7 (63.6)	
N.A.^a^	7	10	3	
Histological type				
Spindle cell	63 (78.8)	30 (68.2)	8 (57.1)	0.160
Other	17 (21.3)	14 (31.8)	6 (42.9)	
N.A.	5	4	0	
Distant metastases during the course of disease				
Absent	73 (85.9)	22 (68.8)	6 (42.9)	0.001
Present	12 (14.1)	15 (31.3)	8 (57.1)	
Tumour necrosis				
Absent	47 (73.4)	22 (71.0)	2 (18.2)	0.001
Present	17 (26.6)	9 (29.0)	9 (81.8)	
N.A.	21	17	3	
Mitotic count (per 50 HPFs)				
0–5	70 (85.4)	31 (66.0)	5 (35.7)	<0.001
>5	12 (14.6)	16 (34.0)	9 (64.3)	
N.A.	3	1	0	
Median age—years (range)	71 (30–92)	68 (44–86)	68 (50–85)	0.191
Median tumour size—cm (range)	5.0 (0.5–33.0)	6.0 (1.5–30.0)	9.0 (1.0–30.0)	0.153
Tumour size class‐cm				
<2	15 (17.6)	6 (12.5)	1 (7.1)	0.501
>2	70 (82.4.)	42 (87.5)	13 (92.9)	

N.A., not available; NIH, the National Institutes of Health; HPF, high‐power field of the microscope.

a GISTs with metastases at the time of the diagnosis (*n* = 20).

When the inter‐rater agreement in immunohistochemical scoring of GIST ITGA4 expression was compared between two independent raters in the western Sweden series, the agreement turned out to be good (kappa coefficient 0.75, 95% CI: 0.65–0.85). The intra‐rater agreement in the western Sweden series was almost perfect (kappa coefficient 0.88, 95% CI: 0.84–0.92).

**Figure 2:**
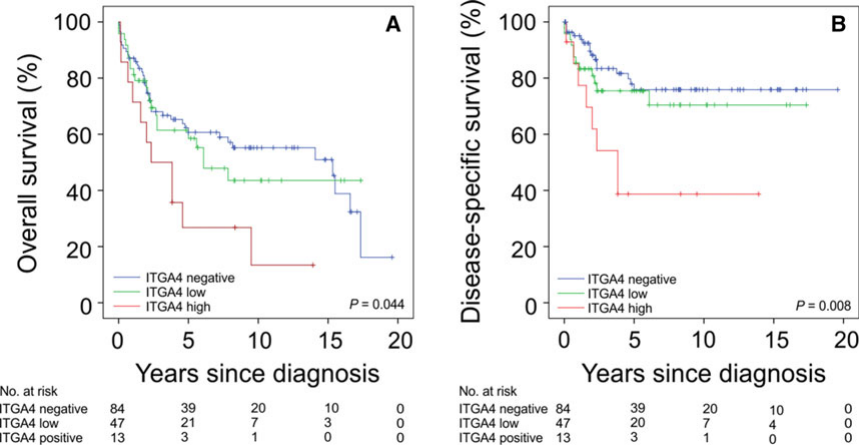
Associations between GIST ITGA4 protein expression with overall survival (**A**) and GIST‐specific survival (**B**). The patients censored are indicated with a bar.

The median follow‐up time of the patients alive was 7.6 years (range, 1–20 years). Seventy‐five (51.0%) patients died during the follow‐up. GIST ITGA4 expression was significantly associated with poor overall survival and GIST‐specific survival (Fig. 2).

### Cell proliferation and invasion analyses

**Figure 3:**
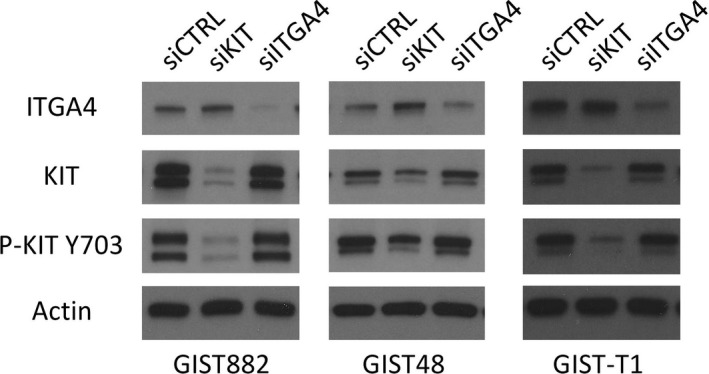
A Western plot showing expression of ITGA4, KIT and actin (control) in the GIST882, GIST48 and GIST‐T1 cell lines. Expression 72 hrs after *KIT* and *ITGA4* siRNA transfection are shown.

**Figure 4:**
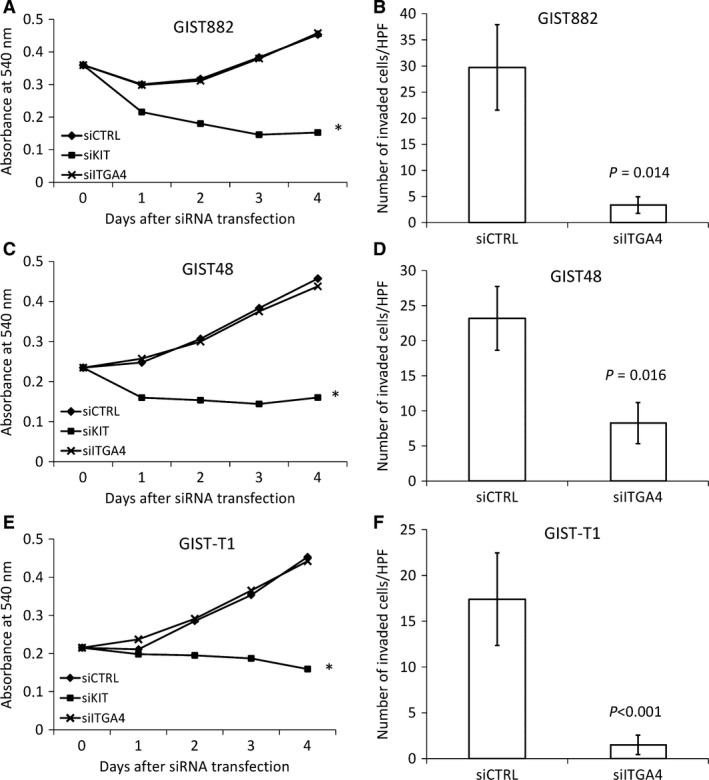
Effect of transfection with control siRNA, *KIT* siRNA and *ITGA4* siRNA on cell proliferation and invasion in GIST882 (**A, B**), GIST48 (**C, D**) and GIST‐T1 (**E, F**) cell lines. Panels (**A, C, E**): cell proliferation decreased with *KIT* siRNA transfection in each cell line, whereas *ITGA4* siRNA transfection had no effect; *P < 0.01. Panels (**B, D, F**): *ITGA4* siRNA transfection decreased cell invasion of all cell lines 24 hrs (GIST882 and GIST48) or 96 hrs (GIST‐T1) after transfection. The bars indicate the average number of invaded cells (the standard errors per one microscope field are shown).

**Figure 5:**
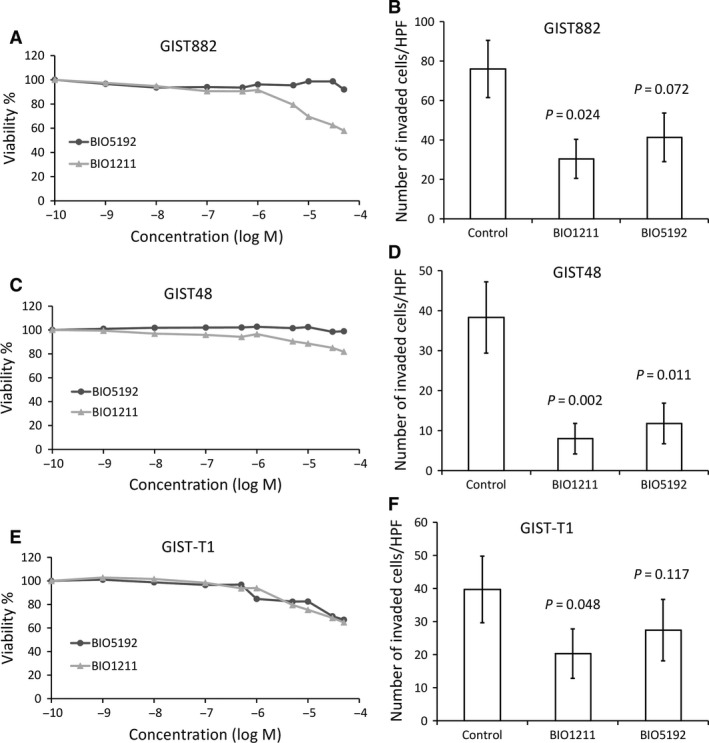
Effect of two VLA‐4 inhibitors (BIO1211 and BIO5192) on cell proliferation and invasion in GIST882 (**A, B**), GIST48 (**C, D**) and GIST‐T1 (**E, F**) cell lines. Panels (**A, C, E**): VLA‐4 inhibitors had little effect on cell proliferation. Panels (**B, D, F**): ITGA4 inhibition with a VLA‐4 inhibitor decreased cell invasion of each cell line 24 hrs (GIST882 and GIST48) or 96 hrs (GIST‐T1) after the treatment. The bars indicate the average number of invaded cells (the standard errors per one microscope field are shown).

As GIST ITGA4 expression was associated with several clinical high‐risk features and unfavourable survival, the function of ITGA4 was further investigated in three GIST cell lines that were all KIT‐positive at immunohistochemistry. ITGA4 protein expression was present in all three cell lines (Fig. 3). When ITGA4 and KIT were depleted in the cell lines with the respective siRNAs, KIT interference with siRNA reduced significantly the proliferation rate of all cell lines during a 4‐day follow‐up period (all *P*‐values <0.001). ITGA4 RNA knock‐down had no effect on the proliferation rate of the cell lines (all *P*‐values >0.05; Fig. 4). VLA‐4 inhibition by BIO1211 and BIO5192 had little effect on cell proliferation (Fig. 5).

The effect of ITGA4 down‐regulation for GIST cell invasion was evaluated in a matrigel‐coated transwell system in the presence of fibronectin in three parallel experiments. The number of invaded cells decreased in the GIST882, GIST48 and the GIST‐T1 cell lines after ITGA4 siRNA transfection as compared with the control siRNAs (GIST882, mean = 3.4, standard error [S.E.] = ±1.6 *versus* control, mean = 29.7, S.E. = ±8.2; *P* = 0.014; GIST48, mean = 8.3, S.E. = ±2.9 *versus* control, mean = 23.2, S.E. = ±4.5, *P* = 0.016; and GIST‐T1, mean = 1.5, S.E. = ±1.1 *versus* control, mean = 17.4, S.E. = ±5.1, *P* < 0.001; Fig. 4).

The VLA‐4‐specific inhibitors had a similar effect to GIST cell invasion as ITGA4‐specific siRNAs. The number of invaded cells decreased in each GIST cell line after VLA‐4 inhibitor treatments. However, only BIO1211 decreased the numbers of invaded cells statistically significantly in the GIST882 and GIST‐T1 cell lines (Fig. 5).

## Discussion

We found that (*i*) ITGA4 is highly expressed in GISTs, (*ii*) decreased ITGA4 signalling blocks GIST cell invasion *in vitro*, (*iii*) ITGA4 protein expression is associated with some adverse prognostic features in GIST such as a high mitotic count, presence of tumour necrosis and metastatic disease and (*iv*) GIST ITGA4 protein expression is associated with unfavourable survival outcome. Somewhat unexpectedly, ITGA4 expression was associated also with gastric location of GIST in the present series, although patients with gastric GIST have, in general, more favourable survival as compared with patients with non‐gastric GIST 31. Little is known about the importance of ITGA4 and other integrins in GIST pathogenesis, but since integrins mediate a wide variety of cellular effects and signalling that can result in tumour progression and metastasis in various types of human cancer 12, it may not be unexpected that integrins are important also in the molecular pathogenesis of GISTs.

To our knowledge, the observations that ITGA4 expression in GISTs is linked with factors such as presence of tumour necrosis, high mitotic counts and poor survival are novel, and they appear to be in line with the reported role of α4 integrins in the embryonal development, normal tissues and in cancer. The α4 integrins are expressed during haematopoiesis on early pluripotent progenitor cells, mononuclear leucocytes and eosinophils 32. In cells such as the myocytes and placental and fibroblastic cells, α4 integrin expression is highly dependent on the developmental stage and growth stimuli 20. α4 integrins have an important role in leucocyte trafficking, and they are involved in the pathogenesis of several chronic diseases including multiple sclerosis, contact hypersensitivity, rheumatoid arthritis and inflammatory bowel diseases 33, 34, 35. ITGA4 expression has been found to be associated with poor prognosis in a few cancer types including neuroblastoma and chronic lymphocytic leukaemia 36, 37, 38. Interaction between ITGA4 in melanoma cells and VCAM‐1 on activated endothelial cells enhances the metastatic capacity of melanoma cells 39, and inhibition of ITGA4 in MV3 melanoma cells with non‐anticoagulant heparin derivatives attenuates melanoma metastatic potential 40. Interestingly, leiomyosarcomas had a similar ITGA4 expression pattern as GISTs. The reasons for this remain speculative.

Besides cell migration and invasion, ITGA4 may also influence cell proliferation. In myeloid cells, ITGA4 activation leads to tumour inflammation and growth, whereas natalizumab, a recombinant humanized anti‐ITGA4 antibody, decreases myeloma growth by blocking the interaction between myeloma cells and bone marrow stromal cells 41. We found a positive association between GIST ITGA4 expression and a high tumour mitotic count in a large GIST patient series, but knock‐down of ITGA4 expression did not influence cell proliferation in GIST cell lines. Tumour ITGA4 expression was associated with poor overall and GIST‐specific survival, and down‐regulation of ITGA4 mRNA expression or VLA‐4 inhibition in cell lines decreased dramatically the invasion activity of GIST cells, suggesting that the major role of ITGA4 in GIST progression is the promotion of tumour invasion and dissemination rather than cell proliferation.

The study has some limitations. Stratifying ITGA4 protein expression based on immunohistochemistry is subjective, but the immunohistochemistry scoring correlated with tumour ITGA4 mRNA qPCR measurements in a subset of cases, and showed associations with clinical parameters in a large clinical GIST series, suggesting that the scoring of the ITGA4 immunostainings reflected the sample ITGA4 content. The somewhat differing proportions of ITGA4‐positive GISTs found in the two series investigated (78.6% and 42.2%) may be due to a higher proportion of low‐risk GISTs in the population‐based western Sweden series, but we cannot exclude the effect of factors that may influence ITGA4 immunostaining, such as differences in tissue sample fixation. Conformation of the current findings in further GIST series is warranted. Study of gene silencing in more than three GIST cell lines could have improved the robustness of the findings, but, to our knowledge, only few GIST cell lines are generally available.

In summary*,* ITGA4 is highly expressed in GISTs as compared with most other human tumour types and most normal tissues. ITGA4 may promote invasiveness of GIST, and GIST ITGA4 protein expression is associated with presence of metastases. Tumour ITGA4 expression was also associated with the high‐risk category of the NIH classification and with poor GIST‐specific and overall survival. The present results suggest that ITGA4 is an important integrin in the molecular pathogenesis of GIST and may influence their clinical behaviour. The findings suggest that agents that target ITGA4 may warrant testing in the treatment of GIST.

## Conflict of interest

The authors have no conflict of interest to declare.

## Supporting information


**Fig. S1** A box‐whisker plot showing the relative *KIT* mRNA expression in histopathologically normal gastric, small intestine, and colorectal tissue (green boxes), and in different types of cancer (red boxes). The number of samples studied is indicated in the brackets. Figure is modified from IST Online^™^ (ist.medisapiens.com).

**Table S1** Comparison between the key GIST prognostic factors in the western Sweden series and in the subgroup of patients included in the current study

**Table S2** Associations between GIST ITGA4 expression and 9 clinicopathological factors in the western Sweden patient series
