# Supplementary figures and images for: Clinical relevance of integrin alpha 4 in gastrointestinal stromal tumours
Source: J Cell Mol Med. 2018 Jan 29;22(4):2220–30. doi: 10.1111/jcmm.13502 (PMC5867167; doi:10.1111/jcmm.13502)

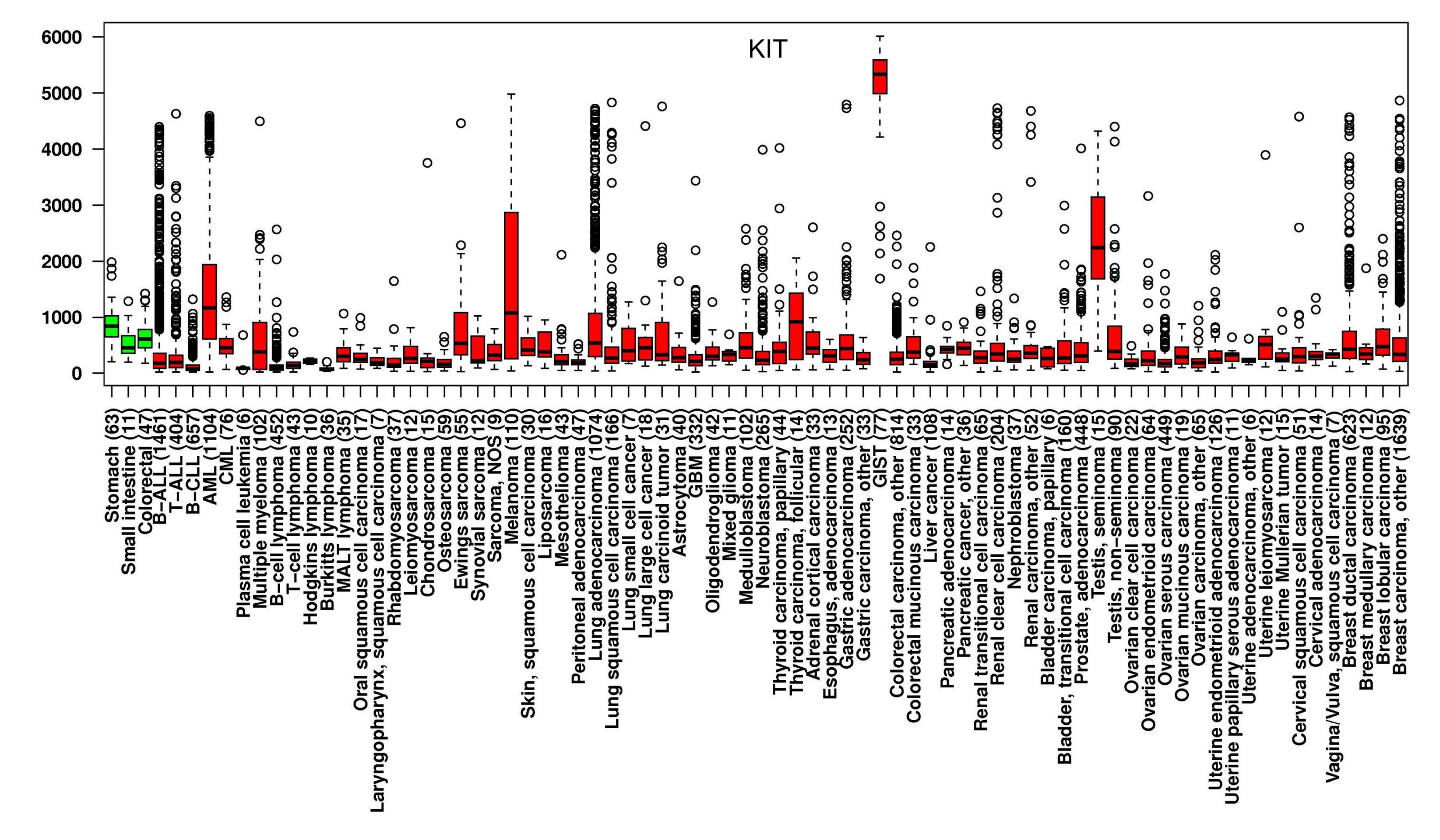

Supplement: Supplementary file 1 — Fig. S1 A box‐whisker plot showing the relative KIT mRNA expression in histopathologically normal gastric, small intestine, and colorectal tissue (green boxes), and in different types of cancer (red boxes). The number of samples studied is indicated in the brackets. Figure is modified from IST Online™ (ist.medisapiens.com). [file JCMM-22-2220-s001.jpg]
