# Supplementary material for: Clinical relevance of integrin alpha 4 in gastrointestinal stromal tumours
Source: J Cell Mol Med. 2018 Jan 29;22(4):2220–30. doi: 10.1111/jcmm.13502 (PMC5867167; doi:10.1111/jcmm.13502)
Supplement: Supplementary file 2 — Table S1 Comparison between the key GIST prognostic factors in the western Sweden series and in the subgroup of patients included in the current study [file JCMM-22-2220-s002.docx]

| **Factor** | **The entire clinical series**  **N = 241**  **n (%)** | **The current study**  **N = 147**  **n (%)** | ***P*** |
| --- | --- | --- | --- |
| Gender |  |  |  |
| Female | 122 (50.6) | 73 (49.7) |  |
| Male | 119 (49.4) | 74 (50.3) | 0.854 |
| Location |  |  |  |
| Gastric | 134 (55.6) | 78 (53.1) |  |
| Non-gastric | 107 (44.4) | 69 (46.9) | 0.238 |
| NIH risk stratification |  |  |  |
| Low/intermediate | 55 (25.5) | 33 (26.0) |  |
| High | 161 (74.5) | 94 (74.0) | 0.915 |
| N.A.* | 25 | 20 |  |
| Mitotic count (per 50 HPFs) |  |  |  |
| 0-5 | 181 (77.4) | 106 (74.1) |  |
| >5 | 53 (22.6) | 37 (25.9) | 0.476 |
| N.A. | 7 | 4 |  |
| Median age - years (range) | 67 (25-92) | 68 (30-92) | 0.800 |
| Median tumour size - cm (range) | 7.4 (0.5-35.0) | 8.1 (0.5-33.0) | 0.095 |

**Supplementary Table 1.** Comparison between the key GIST prognostic factors in the western Sweden series and in the subgroup of patients included in the current study.

Abbreviations: NIH, the National Institutes of Health; N.A., not available; HPF, high-power field of the microscope.
