# Supplementary material for: Clinical relevance of integrin alpha 4 in gastrointestinal stromal tumours
Source: J Cell Mol Med. 2018 Jan 29;22(4):2220–30. doi: 10.1111/jcmm.13502 (PMC5867167; doi:10.1111/jcmm.13502)
Supplement: Supplementary file 3 — Table S2 Associations between GIST ITGA4 expression and 9 clinicopathological factors in the western Sweden patient series [file JCMM-22-2220-s003.docx]

**Supplementary Table 2.** Associations between GIST ITGA4 expression and 9 clinicopathological factors in the western Sweden patient series.

| **Factor** | **GIST ITGA4 expression**  **Negative+Low High**  **N = 133 N = 14**  **n (%) n (%)** | | ***P*** |
| --- | --- | --- | --- |
| Gender |  |  |  |
| Female | 67 (50.4) | 6 (42.9) |  |
| Male | 66 (49.6) | 8 (57.1) | 0.593 |
| Location |  |  |  |
| Gastric | 68 (51.1) | 10 (71.4) |  |
| Non-gastric | 65 (48.9) | 4 (28.6) | 0.148 |
| NIH risk stratification |  |  |  |
| Low/intermediate | 90 (77.6) | 4 (36.4) |  |
| High | 26 (22.4) | 7 (63.6) | 0.007 |
| N.A.* | 17 | 3 |  |
| Histological type |  |  |  |
| Spindle cell | 93 (75.0) | 8 (57.1) |  |
| Other | 31 (25.0) | 6 (42.9) | 0.001 |
| N.A. | 9 |  |  |
| Distant metastases during  the course of disease |  |  |  |
| Absent | 106 (79.7) | 6 (42.9) |  |
| Present | 27 (20.3) | 8 (57.1) | 0.005 |
| Tumor necrosis |  |  |  |
| Absent | 69 (72.6) | 2 (18.2) |  |
| Present | 26 (27.4) | 9 (81.8) | 0.001 |
| N.A. | 38 | 3 |  |
| Mitotic count (per 50 HPFs) |  |  |  |
| 0-5 | 101 (78.3) | 5 (35.7) |  |
| >5 | 28 (21.7) | 9 (64.3) | 0.002 |
| N.A. | 4 |  |  |
| Median age - years (range) | 68 (30-92) | 65 (50-85) | 0.233 |
| Median tumor size - cm (range) | 7.7 (0.5-33.0) | 12.0 (1.0-30.0) | 0.066 |

| Abbreviations: N.A., not available; NIH, the National Institutes of Health; HPF, high-power field of the microscope. | |
| --- | --- |
| *GISTs with metastases at the time of the diagnosis (n = 20). |  |
